# Alcohol consumption: failures and opportunities in the Western Pacific region

**DOI:** 10.1016/j.lanwpc.2025.101489

**Published:** 2025-01-31

**Authors:** The Lancet Regional Health – Western Pacific

In Health Statistics in the Western Pacific Region 2023: Monitoring Health for the SDGs, released on Oct 15, 2024, the WHO Western Pacific region is reported not to be on track to meet the Sustainable Development Goal (SDG) target for non-communicable diseases (NCDs). Smoking and alcohol consumption have significantly contributed to the increase of NCDs and the failure to meet the SDG target. In contrast to the general awareness of the detrimental health effects of smoking, alcohol is not perceived to be as harmful as tobacco.

Scientific evidence suggests alcohol consumption is associated with increased risk of cancer, cardiovascular diseases, injuries, and mental health conditions. Globally, alcohol consumption was linked to 2.6 million deaths in 2019. The higher prevalence of genetic variants of so-called flushing genes, *ALDH2* and *ADH1B*, makes most Asian people less tolerant to alcohol, and east Asia is one of the regions with the highest incidence of alcohol-attributable cancers in the world. Despite these risks, alcohol remains popular in the Western Pacific region, with over 60% of adults consuming 21.3 g alcohol per capita every day.

The popularity of alcohol consumption can be attributed to economic growth, social and cultural norms, along with government support to promote alcohol consumption in the Western Pacific region. It is common for people to drink alcohol either to seek social pleasure, as seen in high-income Australasian countries, or to treat it as a ritual duty in professional settings, promoting social bonding and information sharing with clients and colleagues in east Asian countries, despite no evidence showing that drinking provides advantages in the labour market. Governments quite often play an active role in promoting alcohol consumption for revenue and to attract tourists. For instance, Hong Kong reduced spirits tax from 100% to 10% in October, 2024, and the National Tax Agency of Japan launched a campaign in 2022 to encourage young adults to drink alcohol to generate more tax revenue.

Social norms and misinformation have frequently been used in alcohol marketing to promote drinking by highlighting common behaviours and encouraging doubt and uncertainties about the health risks of alcohol. It is unfair to attribute drinking solely to personal choice, as individuals usually make decisions with imperfect knowledge and limited personal experience. What is unnoticed and untraceable by the public is how alcohol companies lobby and intervene in policy making and implementation. *The Lancet* Series on commercial determinants of health emphasised the central role of reputation management for commercial entities, which enables and interacts with other activities, including policy making, scientific research, marketing, supply chain management, employment, and financial operations. Previous evidence shows minimal health benefits from reputation-building activities from alcohol industries. The goal for profit makes us question if alcohol industries should have a seat at the table in policy making.

The silver lining is that more people are starting to choose moderate or no drinking for health reasons in the Asia–Pacific region. The changing public opinion towards recognising drinking is harmful to health reflects the gradual change of social norms, especially among the younger generation. Despite this, the alcohol market is expected to continue to grow in the Asia–Pacific region, driven by the desire and emergence of online sales.

Raising prices, particularly through taxation, and restricting availability of alcohol are effective policies to curb the growth of alcohol consumption. There are other effective measures, such as mandating label warnings on alcohol products, which is rarely seen in countries in the Western Pacific region. To reduce the rising burden on NCDs, we need stronger political commitment and marketing regulations to nudge the public towards healthier behaviour and reduce the accessibility and affordability of alcohol.

